# Supplementary material for: Vascular Occlusion in Kidney Biopsy Is Characteristic of Clinically Manifesting Thrombotic Microangiopathy
Source: J Clin Med. 2022 May 31;11(11):3124. doi: 10.3390/jcm11113124 (PMC9181253; doi:10.3390/jcm11113124)
Supplement: Supplementary file 1 [file jcm-11-03124-s001.zip › jcm-1671189-supplementary.pdf]

Supplementary Table S1. Univariate and multivariate analysis of histological changes predicting progression in whole study population.

Variable	Whole population					
	Univariate			Multivariate		
	OR	95% CI	p-value	OR	95% CI	p-value
Mesangiolysis	10.0	1.34-74.51	0.025	0.03	0 - inf	1.000
Glomerular thrombi	5.85	1.06-32.1	0.042	3.78e+27	0 (NA)	0.999
Severe doubled GBM	0.07	0.01-0.82	0.035	1.04e-18	0 (NA)	0.999
Arterial myxoid swelling	13.5	1.95-93.24	0.008	7.84e-12	0 (NA)	1.000
”Onion” skinning	10.0	1.34-74.51	0.025	2.29e+18	0 (NA)	0.999
Podocyte effacement	1.06	1.01-1.11	0.018	1.03	0.95 – 1.12	0.472

GBM=glomerular basement membrane, NA = not applicable
